# The Impact of Pulmonary Rehabilitation in Patients with Post-COVID-19 Syndrome: A Systematic Review

**DOI:** 10.3390/medsci14040443

**Published:** 2026-07-27

**Authors:** Vlad-Florin Oiegar, Cristina Călărașu, Paraschiva Postolache, Constantin Ghimuș, Mara-Amalia Bălteanu, Simona Pătru, Dănuț Caimac, Ana Maria Bumbea, Simona-Maria Roșu, Ionela Alina Croitoru

**Affiliations:** 1 Doctoral School, Grigore T. Popa University of Medicine and Pharmacy, 700115 Iași, Romania; oiegar.vlad-florin@d.umfiasi.ro (V.-F.O.); cosghimus@gmail.com (C.G.); 2 Department of Biomedical Sciences, Faculty of Medical Bioengineering, Grigore T. Popa University of Medicine and Pharmacy, 700115 Iași, Romania; 3 Medical Department 3, University of Medicine and Pharmacy, 200349 Craiova, Romania; calarasu.cristina@yahoo.com; 4 Academy of Romanian Scientists, 050044 Bucharest, Romania; 5 Department of Pulmonology, Faculty of Medicine, Titu Maiorescu University, 031593 Bucharest, Romania; mara.balteanu@prof.utm.ro; 6 Department of Pneumology, “Marius Nasta” Institute for Pneumology, 050159 Bucharest, Romania; alina.haulinca@umfcd.ro; 7 Medical Rehabilitation Department, University of Medicine and Pharmacy, 200349 Craiova, Romania; simona.patru@umfcv.ro (S.P.); anamaria.bumbea@umfcv.ro (A.M.B.); simona.rosu@umfcv.ro (S.-M.R.); 8 Doctoral School, University of Medicine and Pharmacy, 200349 Craiova, Romania; 9 Faculty of Medicine, Carol Davila University of Medicine, 050474 Bucharest, Romania

**Keywords:** exercise capacity, muscle function, dyspnea, quality of life, rehabilitation protocols, telerehabilitation

## Abstract

Background: Post-COVID-19 syndrome represents a global health problem, manifesting with persistent multiple organ symptoms, including respiratory ones. This review aimed to evaluate the efficacy of different pulmonary rehabilitation interventions in the management of the disease, including its particularities. Methods: This systematic review was conducted in accordance with the Preferred Reporting Items for Systematic Reviews and Meta-Analyses (PRISMA 2020) statement and prospectively registered in the PROSPERO database (CRD420261292824). PubMed, Embase, and the Cochrane Central Register of Controlled Trials were searched from January 2021 to January 2026. Two reviewers independently screened studies, extracted data, and assessed methodological quality using the Cochrane Risk of Bias 2 (RoB 2) tool. Twenty randomized controlled trials involving 2101 adult patients with post-COVID-19 syndrome met the eligibility criteria and were included in the qualitative synthesis. Results: Pulmonary rehabilitation (PR) demonstrated significant improvements in exercise capacity, with notable increases in 6MWT and VO_2_max. Respiratory and peripheral muscle strength significantly increased. The quality of life, dyspnea and fatigue consistently improved. The results for intrinsic pulmonary function, anxiety, and depression were heterogeneous, suggesting benefits more related to reconditioning. Conclusions: Pulmonary rehabilitation is an essential and efficient component in post-COVID-19 syndrome, improving physical function, respiratory and peripheral muscle strength, the quality of life, fatigue and dyspnea. Telerehabilitation is a viable alternative. Adapting protocols is crucial, recognizing that most benefits derive from physical reconditioning and muscle training, not necessarily from major structural pulmonary changes.

## 1. Introduction

Post-COVID-19 syndrome is characterized by symptoms that appear 3 months after SARS-CoV-2 virus infection, and last at least two months. Post-COVID-19 syndrome represents a frequent global health problem, with a global prevalence of 6%, according to the World Health Organization [1]. The risk factors for developing the syndrome include repeated COVID-19 infections, severe COVID-19 disease (requiring hospitalization/ICU admission), female sex, active smoker status, advanced age, overweight/obesity, comorbidities (chronic pulmonary diseases, chronic kidney disease, type 2 diabetes mellitus, connective tissue diseases), and melatonin deficiency. The risk of developing post-COVID-19 syndrome is lower in patients previously immunized with two doses of the vaccine (mRNA, viral vector, or combination) against SARS-CoV-2 [2].

### 1.1. Pathogenesis

The pathophysiological mechanism is not fully elucidated, but several pathogenic pathways interact and contribute to the onset of symptoms. Immune dysregulation can be caused by the persistence of the SARS-CoV-2 virus or the reactivation of other viruses (EBV, HSV6), intestinal dysbiosis, abnormal neurological signaling, autoimmunity, endothelial dysfunction, and hypercoagulability. A hyperinflammatory response, partially facilitated by mast cell activation, is linked to a decrease in the number of B and T lymphocytes and an increase in innate inflammation, further contributing to the chronic inflammatory response and immune activation in post-COVID-19 syndrome. An insufficient immune response during the acute phase of COVID-19 may contribute to virus persistence (spike protein and mRNA) in tissue reservoirs, and may trigger a repeated and sustained immune response.

SARS-CoV-2 infection, causing intestinal dysbiosis, increases intestinal permeability and the translocation of pathogenic agents into circulation, with implications for chronic inflammation persistence and endothelial dysfunction. Endothelial dysfunction, altered cell morphology, and even cell apoptosis contribute to the hypercoagulable state characteristic of COVID-19, leading to microscopic hemorrhages, microclots, and ischemic lesions in all territories and organs, predominantly affecting the brain and heart [3,4,5].

Metabolic and hormonal imbalances (hypothalamic–pituitary–adrenal axis dysfunction) characterize post-COVID-19 syndrome. Hypophysitis, hypothalamic lesions, neuroinflammation, or molecular mimicry (ACTH residue) may be associated with neurotoxicity, neurodegeneration, depression, neurocognitive disorders, and autonomic nervous system dysfunction. The molecular mechanism of impaired concentration involves increased levels of AMPA receptors (AMPARs), important molecules in learning and memory processes. AMPAR levels have been directly correlated with the severity of lack of concentration and increased inflammatory markers [6].

The metabolic impairment in SARS-CoV-2 is also expressed as an alteration of glucose homeostasis. Elevated serum glucose levels have been correlated with markers of inflammation (erythrocyte sedimentation rate (ESR) and C-reactive protein (CRP)), but also with the presence of respiratory failure and increased mortality rates [7].

### 1.2. Pathophysiology of Exercise Intolerance and Respiratory Dysfunction

The pathophysiological mechanisms driving post-COVID-19 syndrome are multifaceted, shifting from acute viral injury to chronic structural and functional abnormalities that directly impair physical activity, exercise capacity, and respiratory function. While immune dysregulation, persistent viral reservoirs, and endothelial injury initiate the syndrome, the resultant functional decline is primarily mediated by objective cardiopulmonary alterations, autonomic dysregulation, and peripheral muscle dysfunction.

### 1.3. Cardiopulmonary Exercise Characteristics

Cardiopulmonary exercise testing (CPET) in post-COVID-19 patients consistently reveals a marked reduction in maximal oxygen uptake VO_2_max and an early anaerobic threshold (AT). This objective exercise intolerance is frequently accompanied by ventilatory inefficiency, evidenced by an elevated VE/VCO_2_ slope, which suggests alveolar–capillary membrane diffusion defects, microvascular microclots, or ventilation–perfusion (V/Q) mismatching. Furthermore, chronotropic incompetence is a prominent feature limiting peak workload during functional assessments [8].

#### 1.3.1. Autonomic Dysfunction

Autonomic nervous system dysregulation plays a critical role in the pathogenesis of exercise intolerance and post-exertional malaise (PEM). Conditions such as postural orthostatic tachycardia syndrome (POTS) and inappropriate sinus tachycardia are highly prevalent in this population. This autonomic mismatch disrupts normal cardiovascular hemodynamics during exertion, leading to altered heart rate variability (HRV), impaired heart rate recovery post-exercise, and inappropriate regional blood flow distribution. Consequently, perfusion to both central respiratory centers and peripheral locomotive muscles is compromised, exacerbating dyspnea and functional limitations [9].

#### 1.3.2. Skeletal Muscle Endurance and Deconditioning

Beyond central cardiopulmonary constraints, peripheral structural adaptations heavily compromise skeletal muscle endurance. Persistent systemic inflammation and microvascular endothelial dysfunction induce localized tissue hypoxia and capillary rarefaction within skeletal muscle beds. Emerging evidence highlights mitochondrial dysfunction as a primary driver of myalgia and reduced muscle endurance [8,10].

### 1.4. Clinical Presentation

The disease is multisystemic, with clinical impact on the lungs, heart and blood vessels, nervous system, digestive, reproductive, excretory, ENT, and endocrine systems (Table 1). Over 200 symptoms have been described by patients, the most frequent being as follows: dyspnea, fatigue, arthralgia, myalgia, headache, difficulty concentrating and thinking, and dysgeusia. Some patients suffering from post-COVID-19 syndrome experience post-exertional malaise (PEM), which represents an accentuated alteration of the general state and symptomatology after performing low-intensity physical/mental effort. Triggering events can be daily activities, wide variations in sound or light, and agitated environments. The reaction can be immediate, or it appears 24–72 h after effort, potentially lasting for days or even weeks [11]. Some patients may present with dyspnea, a dry, nagging cough and severe, lingering fatigue, symptoms that accompany pulmonary fibrosis, which most frequently develops in 20–70% of the patients who required intensive care or mechanical ventilation during the acute phase of the infection [12,13].

### 1.5. Physiological and Functional Parameters During Physical Activity

To optimize pulmonary rehabilitation strategies, the clinical presentation of post-COVID-19 syndrome must be understood through objective physiological impairments observed during physical exertion, rather than relying solely on subjective patient-reported symptoms. Persistent functional limitations in these patients are characterized by distinct abnormalities in pulmonary, cardiovascular, and metabolic responses to exercise.

### 1.6. Pulmonary Function and Lung Volumes

At rest, standard pulmonary function parameters frequently reveal a persistent reduction in the diffusing capacity of the lungs for carbon monoxide (TLCO), signaling alveolar–capillary membrane disruption. However, advanced plethysmographic assessments also demonstrate significant alterations in lung volumes, specifically an elevated residual lung volume (RV) and RV/total lung capacity (TLC) ratio in a subset of patients. During physical activity, these baseline restrictions compromise ventilatory efficiency, leading to an abnormal increase in minute ventilation (VE) relative to carbon dioxide production (VE/VCO_2_ slope). This ventilatory mismatch directly drives the severe, disproportionate exertional dyspnea experienced by these patients [14].

### 1.7. Aerobic and Anaerobic Exercise Capacity

Cardiopulmonary exercise testing (CPET) objectively quantifies a marked reduction in overall aerobic capacity, demonstrated by a significantly lower peak oxygen consumption (VO_2_max) and a premature reach of their anaerobic threshold (AT) during incremental exercise. Consequently, both aerobic and anaerobic exercise capacities are severely blunted, restricting the patient’s ability to perform activities of daily living without experiencing profound fatigue [15].

### 1.8. Cardiovascular Dynamics and Heart Rate Reserve

Cardiovascular response to physical exertion is characterized by autonomic dysregulation. Patients frequently exhibit a severely reduced heart rate reserve alongside chronotropic incompetence and delayed heart rate recovery post-exercise. This autonomic blunting prevents appropriate hemodynamic scaling during exercise, leading to inappropriate cardiac output and regional tissue hypoperfusion [16].

Targeting these objective cardiorespiratory and metabolic deficits is central to pulmonary rehabilitation. The clinical improvements are directly mediated by the physiological restoration of muscle metabolic pathways, improved ventilatory efficiency, and the stabilization of autonomic cardiovascular responses, underscoring the necessity of exercise-based interventions in restoring functional independence.

### 1.9. Treatment of Post-COVID-19 Syndrome: The Key Role of Pulmonary Rehabilitation

The management of post-COVID-19 syndrome requires a multidisciplinary team consisting of physical and rehabilitation medicine physician, pulmonologist, cardiologist, general practitioner, physiotherapist, psychologist, nutritionist, occupational therapist, and, depending on the heterogeneity of symptoms: neurologist, psychiatrist, nephrologist, hematologist [17]. Pharmacological treatment includes symptomatic treatment, therapy with antifibrotic agents (in cases of proven fibrosis), and vaccination against SARS-CoV-2. Current studies suggest that the vaccine may have protective and therapeutic effects on patients. Routine use of anticoagulants is not recommended [18,19].

Pulmonary rehabilitation, defined according to the 2023 American Thoracic Society (ATS) guidelines, is a set of multidimensional interventions, designed with a personalized approach, aiming to improve the physical and psychosocial well-being of patients with chronic respiratory diseases [20]. Pulmonary rehabilitation represents the cornerstone in the management of post-COVID-19 syndrome, with proven benefits through systematic reviews regarding symptom improvement, increased exercise capacity, the optimization of pulmonary function, and improved quality of life [21,22,23]. However, the correlation between rehabilitation interventions and certain outcomes, such as pulmonary function, fatigue, and quality of life, has shown inconsistencies in these studies.

### 1.10. Structured Exercise-Based Rehabilitation Protocols

To maximize functional recovery, and address the objective cardiopulmonary and musculoskeletal deficits of post-COVID-19 syndrome, rehabilitation programs must transition from general physical activity recommendations to structured, individualized exercise prescriptions.

### 1.11. Endurance (Aerobic) Training Modalities

Continuous or interval-based training utilizes stationary cycling (vertical or recumbent to accommodate autonomic instability), treadmill walking, or arm ergometry. The intensity should be kept within a light-to-moderate range, starting with short, manageable intervals of 10 to 15 min, gradually progressing to 30 to 45 min of continuous exercise as tolerated [24].

### 1.12. Resistance (Strength) Training Modalities

Resistance training targets peripheral muscle weakness, reverses muscle wasting (sarcopenia) secondary to acute-phase immobilization, and improves mechanical efficiency during daily tasks. Progressive overload exercises using body weight, elastic resistance bands, light free weights (dumbbells), or specialized weight machines should be used for 2 to 3 non-consecutive days per week to allow for adequate muscular recovery [25].

### 1.13. Inspiratory Muscle Training (IMT)

Given the high prevalence of diaphragmatic dysfunction and ventilatory inefficiency in post-COVID-19 patients, targeted respiratory training is essential. Threshold or resistive inspiratory muscle training devices should be used daily, consisting of 2 sets of 30 breaths or approximately 15 to 20 min of cumulative training per day. 

A crucial safeguard in post-COVID-19 rehabilitation is the prevention of post-exertional malaise (PEM), a severe worsening of symptoms after minor physical or mental exertion. Exercise sessions must be immediately adjusted or paused if patients experience oxygen desaturation (SpO_2_ < 90%), inappropriate tachycardia, or extreme fatigue persisting more than 24 h post-exercise [26]. Exercise pacing and heart rate monitoring are vital tools used to keep patients within their aerobic energy pathways, avoiding the anaerobic threshold where metabolic dysregulation occurs.

Given the publication of several new systematic reviews and meta-analyses [27,28,29,30], it is essential to update the available scientific evidence on this topic to evaluate the effect of pulmonary rehabilitation on symptoms, pulmonary function, and respiratory and peripheral muscle strength in patients with post-COVID-19 syndrome. In this context, the objective of this review was to comprehensively evaluate the effects of pulmonary rehabilitation in patients with post-COVID-19 symptoms.

## 2. Methodology

### 2.1. Study Design, Registration, and Objectives

This study is a systematic review developed in accordance with the Preferred Reporting Items for Systematic Reviews and Meta-Analysis (PRISMA), and was registered before starting in PROSPERO with ID code number CRD420261292824.

This systematic review, conducted between January and February 2026, aimed to provide an update about the efficacy of pulmonary rehabilitation in patients with post-COVID-19 syndrome. The first objective was to evaluate the impact of different pulmonary rehabilitation approaches on the symptoms, pulmonary function, exercise capacity, peripheral muscle performance, respiratory muscle function and the quality of life in patients with post-COVID-19 syndrome. The second objective was to assess the importance and feasibility of pulmonary rehabilitation delivered via telerehabilitation (tele-PR) compared to in-hospital PR.

### 2.2. Search Strategy

The search strategy combined controlled vocabulary (Medical Subject Headings [MeSH]) and free-text terms related to post-COVID-19 syndrome and pulmonary rehabilitation. The complete PubMed search strategy was as follows: (“COVID-19”[Mesh] OR “Long COVID” OR “Post-COVID syndrome” OR “Post-COVID-19 condition”) AND (“Pulmonary Rehabilitation” OR “Exercise Therapy” OR “Respiratory Rehabilitation” OR “Exercise Training”). The following filters were applied:

- Humans;
- Adults (≥18 years);
- English language;
- Randomized controlled trial;
- Publication period: January 2021 to January 2026.

Equivalent search strategies adapted to the indexing systems of Embase and the Cochrane Central Register of Controlled Trials were also used. Full-text articles were obtained from databases from 2021 up to January 2026.

The electronic databases were last searched on 31 January 2026. Additionally, the reference list of all eligible randomized controlled trials and relevant systematic reviews were manually screened to identify any potentially eligible studies not retrieved through the electronic search. Grey literature, conference abstracts, preprints, and unpublished studies were not included in the present review.

### 2.3. Study Screening

All records retrieved through the electronic search were imported into a reference management software, and duplicate records were removed before screening. Two reviewers (V.-F.O. and C.G.) independently screened the titles and abstracts to identify potentially eligible studies. Full-text articles were subsequently assessed independently according to the predefined eligibility criteria. Any disagreements regarding study eligibility were resolved through discussion and consensus with a third reviewer (P.P.). No automation tools or artificial intelligence-assisted screening software were used during the selection process.

### 2.4. Eligibility Criteria

The inclusion criteria for the studies were as follows: (a) randomized clinical trials (RCTs) that included adult patients (>18 years) diagnosed with post-COVID-19 syndrome who had confirmed COVID-19 disease, from inception to January 2026; (b) RCTs that had the following (primary or secondary) parameters as objectives: exercise capacity, pulmonary function, respiratory muscle function, the quality of life, dyspnea, fatigue, peripheral muscle strength, anxiety, depression; and (c) RCTs that compared in-hospital PR group with a group that received PR via telerehabilitation, used a sham device, standard care, educational counseling, or which received no treatment. The inclusion criteria were applied by two of the researchers (A.M.B. and S.M.R.) and disagreements were resolved through consensus with the other author (V.F.O.).

We excluded observational studies, non-randomized controlled trials, systematic reviews, meta-analysis, case report studies, editorials, abstracts, letters, protocols, and duplicates of the included studies. Studies were excluded if they included patients with positive COVID-19 (acute disease), or subacute disease or included patients that did not receive pulmonary rehabilitation. The final decision to include or exclude a study was based on a full-text review, focusing on the study type, objectives, and results.

For the purpose of the qualitative synthesis, the included studies were grouped according to the rehabilitation delivery modality (face-to-face pulmonary rehabilitation versus telerehabilitation), intervention characteristics (exercise training, inspiratory muscle training, respiratory muscle training, multicomponent rehabilitation, or combined interventions), and the primary clinical outcomes evaluated.

### 2.5. Quality and Risk of Bias Evaluation

The risk of bias of the articles that were included in the review was assessed using the Cochrane Risk of Bias Assessment Tool for Randomized Controlled Trials (RoB 2.0) which contains five domains: bias arising from the randomization process, bias due to deviation from intended interventions, bias due to missing outcome data, bias in the measurement of the outcome, and bias in the selection of the reported result. Two of the researchers (A.M.B and I.A.C) independently assessed the methodological quality. In the case of any doubts or disagreements, they were resolved through consensus with another author (V.F.O). Each domain was scored as “low risk”, “some concerns”, and “high risk”, and each study was classified into one of three categories as “high risk of bias”, “some concerns”, or “low risk of bias”.

To evaluate the methodological quality of the included clinical trials, we utilized the Physiotherapy Evidence Database (PEDro) scale (Table 2). The PEDro scale is a specialized, internationally recognized instrument designed specifically for physical therapy and rehabilitation trials. It allows readers to rapidly identify whether a trial has sufficient internal validity and statistical information to make its clinical results interpretable. The PEDro scale consists of 11 items, scored as either “Y-yes” (1 point) or “N-no” (0 point) based on whether the study meets the specified criteria. Item 1 assesses external validity (eligibility criteria) but is excluded from the final total score, items 2–9 evaluate internal validity (including random allocation, concealed allocation, baseline similarity, and blinding of subjects, therapists, and assessors), and items 10–11 assess the adequacy of statistical reporting (intention-to-treat analysis, between-group comparisons, and point measures/variability) [31].

### 2.6. Data Extraction

Data extraction was independently performed by reviewers (V.-F.O., P.P., C.G., S.P.) using a standardized data extraction form specifically developed for this review. The extracted information included study characteristics, participant demographics, severity of the acute COVID-19 episode, pulmonary rehabilitation protocol, comparator intervention, duration of treatment, outcome measures, and statistically significant intragroup and intergroup findings. Any discrepancies between reviewers were resolved through discussion and consensus with a third reviewer (P.P.). The authors of the included studies were not contacted for additional information because all relevant data required for the qualitative synthesis were available in the published articles.

Studies were grouped according to rehabilitation modality (hospital-based pulmonary rehabilitation versus telerehabilitation), intervention characteristics, and clinical outcomes in order to facilitate the qualitative synthesis. No imputation of missing data was performed. Only data explicitly reported in the original publications were extracted and synthesized. The study characteristics and outcome data were summarized using structured evidence tables, while the overall findings were synthesized narratively according to each predefined outcome. A quantitative meta-analysis was not performed because of substantial clinical heterogeneity among studies regarding rehabilitation protocols, intervention duration, exercise intensity, outcome measures, comparator groups, and follow-up periods. Subgroup analyses or meta-regression were not feasible because no quantitative synthesis was performed. 

### 2.7. Data Analysis

The primary outcomes of this systematic review were exercise capacity, pulmonary function, and respiratory muscle function. Exercise capacity was assessed using validated measures including the six-minute walk test (6MWT), cardiopulmonary exercise testing (CPET), maximal oxygen uptake (VO_2_max), and incremental shuttle walk test (ISWT). Pulmonary function outcomes included spirometric parameters (FEV_1_, FVC, FEV_1_/FVC) and other respiratory function measurements when available. Respiratory muscle function was evaluated using maximal inspiratory pressure (MIP), maximal expiratory pressure (MEP), sniff nasal inspiratory pressure (SNIP), or equivalent validated measures.

The secondary outcomes included peripheral muscle strength, dyspnea, fatigue, health-related quality of life, anxiety, depression, rehabilitation adherence, intervention duration, rehabilitation setting (hospital-based or telerehabilitation), severity of the acute COVID-19 episode, age, sex, and country of origin.

Because the included studies reported outcomes using different measurement instruments and scales, and substantial clinical heterogeneity was identified across rehabilitation protocols and comparators, pooled quantitative effect estimates were not calculated. Treatment effects were, therefore, summarized descriptively according to statistically significant intragroup and between-group differences reported by the original studies.

### 2.8. Assessment of Reporting Bias

Reporting bias was not formally assessed because no quantitative meta-analysis was performed and fewer than ten studies contributed to each individual outcome. Therefore, funnel plots and statistical tests for publication bias were not considered appropriate.

### 2.9. Certainty of the Evidence

The certainty of the evidence was not formally assessed using the GRADE approach because the objective of this review was to provide a qualitative synthesis of randomized controlled trials, and substantial clinical heterogeneity precluded quantitative pooling of the results.

## 3. Results

### 3.1. Study Selection

**Figure 1:**
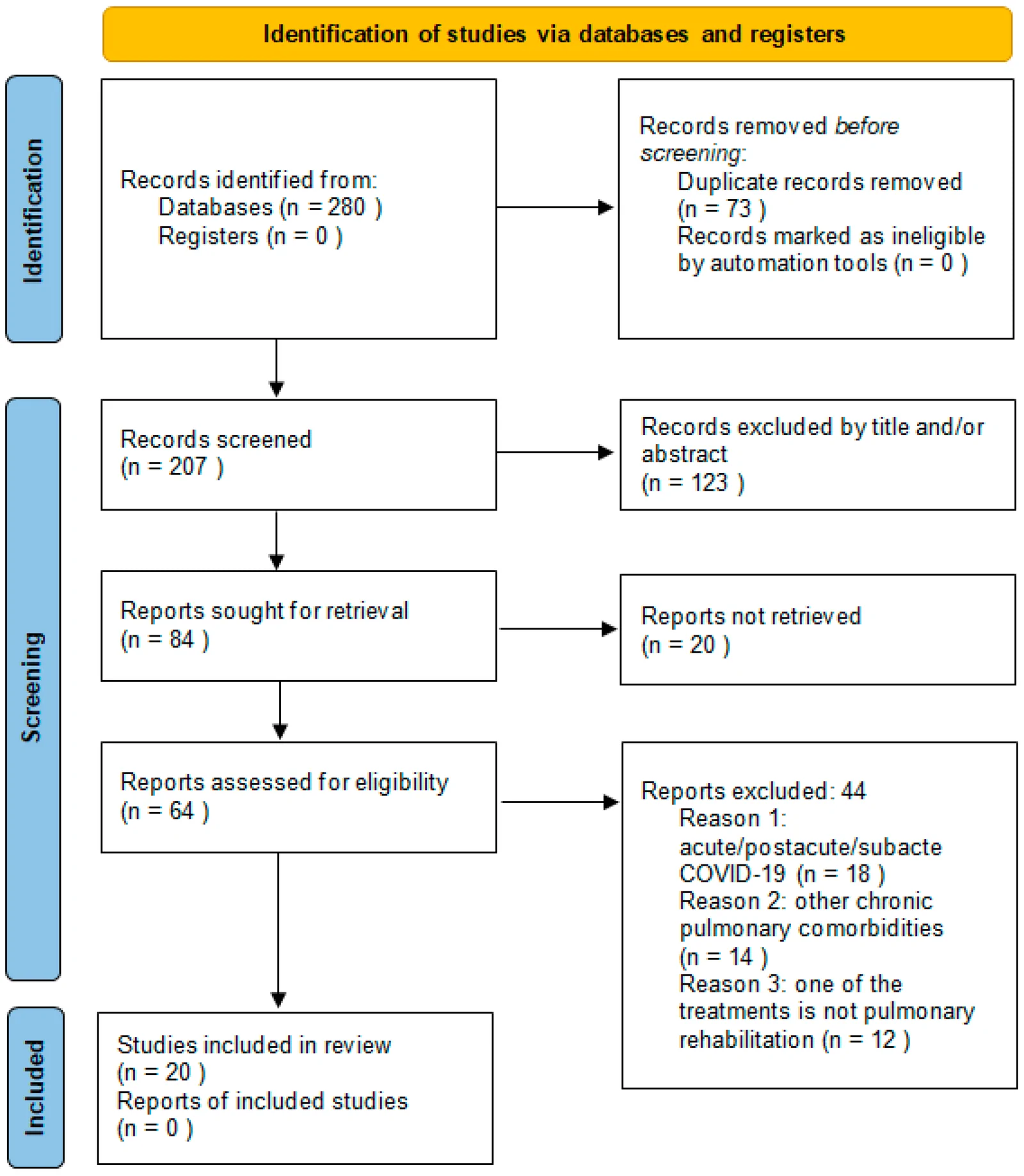
Descriptive flow diagram for the study selection.

By following the four-stage PRISMA flow diagram, which details the identification, screening, eligibility, and ultimate inclusion of studies, we minimized reporting bias and ensured a systematic, non-biased approach to the literature selection [32]. After the search strategies were implemented, a total of 280 articles were found. After removing the duplicates, the number was narrowed down to 207. Upon reviewing the titles and/or abstracts, a total of 123 articles were excluded, narrowing the number of records sought for retrieval to 84. Twenty reports could not be retrieved and subsequently, a full reading of the 64 retrieved articles was conducted for a more in-depth evaluation. In the end, a total of 20 studies which met the eligibility criteria were included. The detailed selection process for the articles included in this review can be found in Figure 1.

### 3.2. Methodological Quality

According to the PEDro scale [33,34,35,36,37,38,39,40,41,42,43,44,45,46,47,48,49,50,51,52], sixteen articles [33,34,35,36,37,38,39,40,42,43,44,45,49,50,51,52] received a score of 6–8/10, indicating that the studies were designed with good methodological quality. According to the scale, two articles written by the same first author [41,48] have an excellent methodological quality (score 9–10/10) and only two [46,47] scored 5/10, indicating a fair methodological quality. 

**Table 2:** Methodological quality according to the PEDro scale.

Study No. and First Author	Q1	Q2	Q3	Q4	Q5	Q6	Q7	Q8	Q9	Q10	Q11	Total Score
[33]: Longobardi et al.	Y	Y	Y	Y	N	N	Y	N	Y	Y	Y	7/10
[34]: Daynes et al.	Y	Y	Y	N	N	N	Y	N	Y	Y	Y	6/10
[35]: Rutkowski et al.	Y	Y	Y	Y	N	N	Y	Y	N	Y	Y	7/10
[36]: Sánchez Milá et al.	Y	Y	Y	Y	N	N	Y	Y	N	Y	Y	7/10
[37]: McNarry et al.	Y	Y	Y	Y	N	N	N	N	Y	Y	Y	6/10
[38]: Okan et al.	Y	Y	Y	Y	N	N	N	Y	Y	Y	Y	7/10
[39]: Philip et al.	Y	Y	Y	Y	N	N	Y	Y	Y	Y	Y	8/10
[40]: McGregor et al.	Y	Y	Y	Y	N	N	Y	Y	Y	Y	Y	8/10
[41]: del Corral et al.	Y	Y	Y	Y	Y	Y	Y	Y	Y	Y	Y	10/10
[42]: Elyazed et al.	Y	Y	Y	Y	N	N	N	Y	N	Y	Y	6/10
[43]: dos Santos et al.	Y	Y	Y	N	N	N	Y	Y	N	Y	Y	6/10
[44]: Jimeno-Almazan et al.	Y	Y	Y	Y	N	N	N	Y	N	Y	Y	6/10
[45]: Li et al.	Y	Y	Y	Y	N	N	Y	Y	Y	Y	Y	8/10
[46]: Vallier et al.	Y	Y	Y	Y	N	N	N	N	N	Y	Y	5/10
[47]: Arora et al.	Y	Y	Y	Y	N	N	N	N	N	Y	Y	5/10
[48]: del Corral et al.	Y	Y	Y	Y	Y	Y	Y	Y	Y	Y	Y	10/10
[49]: Nagy et al.	Y	Y	Y	Y	N	N	Y	Y	Y	Y	Y	8/10
[50]: Romanet et al.	Y	Y	Y	Y	N	N	Y	Y	Y	Y	Y	8/10
[51]: Bai et al.	Y	Y	Y	Y	N	N	N	Y	Y	Y	Y	7/10
[52]: Besnier et al.	Y	Y	Y	Y	N	N	Y	Y	Y	Y	Y	8/10

### 3.3. Risk of Bias Assessment

**Figure 2:**
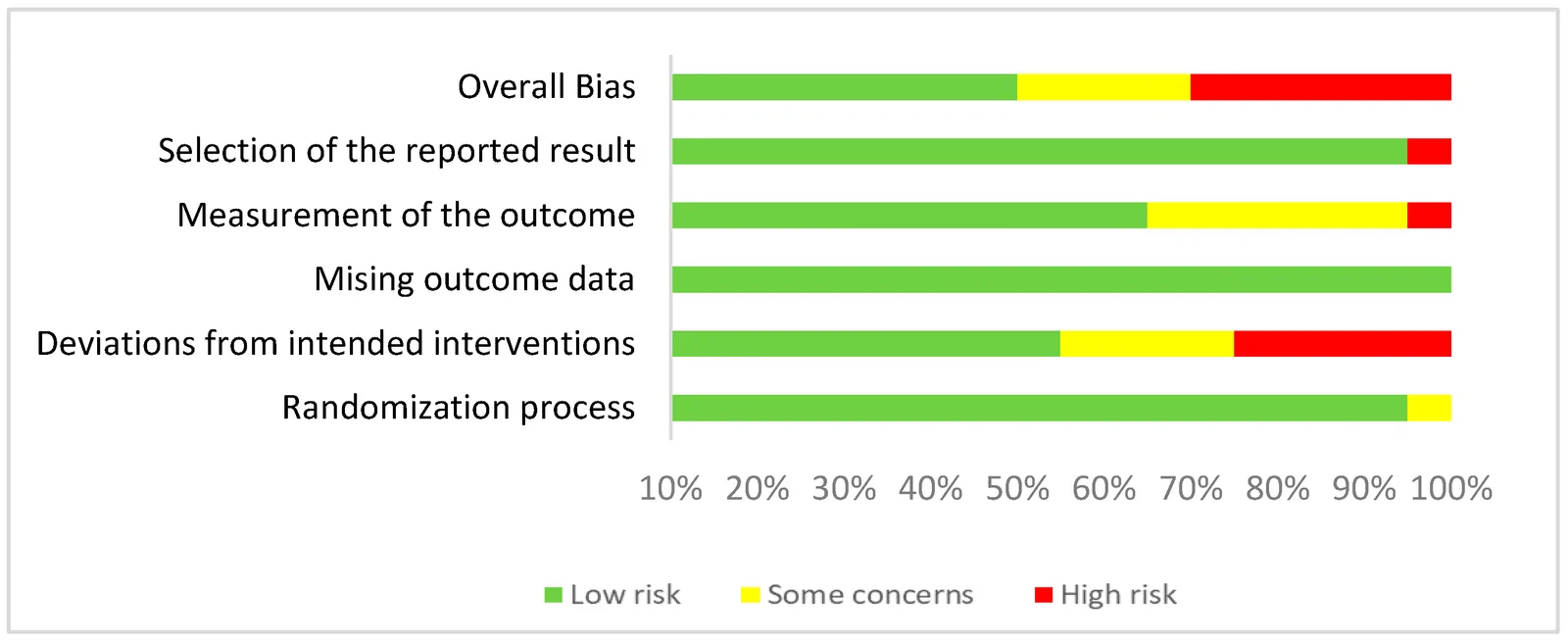
Risk of bias graph.

**Figure 3:**
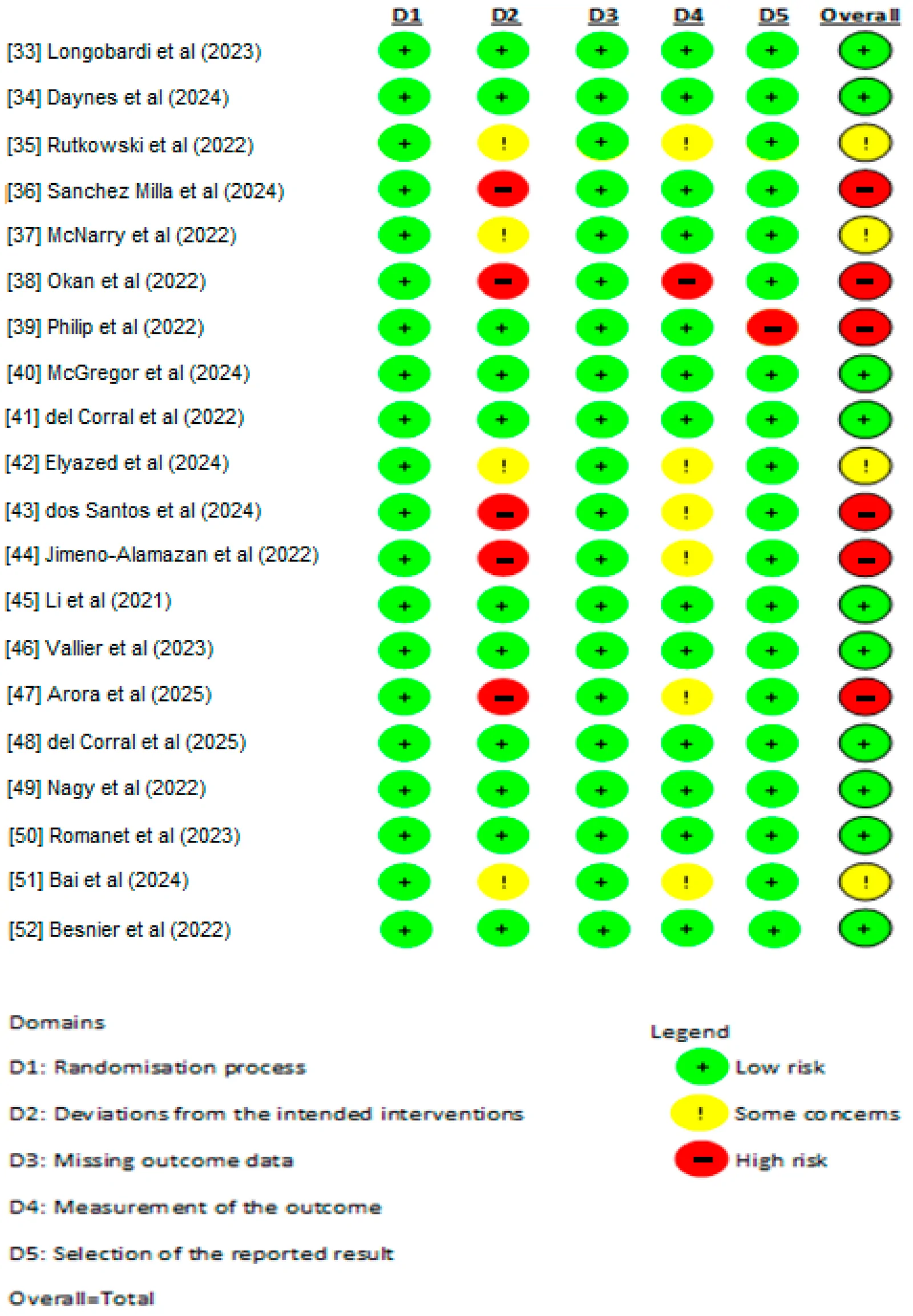
Risk of bias summary.

The risk of bias of RCTs ranged from low to high, with ten studies with a low risk of bias [33,34,40,41,45,46,48,49,50,52], four with some concerns [35,37,42,51], and six with a high risk [36,38,39,43,44,47]. Bias due to deviation from the intended interventions was the domain with higher issues, while the domains related to the randomization and missing outcome data were the domains with the best scores. The quality of evidence of RCTs can be found in Figure 2 and Figure 3.

### 3.4. Characteristics of Participants and Intervention

The results of each included randomized controlled trial were extracted and synthesized according to the predefined outcomes. The outcome measures, intervention characteristics, and statistically significant intragroup and between-group differences are summarized in Table 3 and Table 4.

A total number of 1915 patients (56,24% female), aged between 18 and 87 years, with post-COVID-19 syndrome were included in the 20 studies that met the eligibility criteria. The RCT, with the largest sample size, included a total of 485 participants [40] and the study with the fewest sample size involved a total of 17 individuals [46]. Half of the RCTs [33,35,37,38,39,40,42,45,46,47] compared tele-PR with in-hospital PR or usual care.

Most of the included studies carried out performed PR programs based on exercise and breathing retraining as the main components, varying in the number of sessions and intervention approaches employed. Strength training, in addition to aerobic exercise, was performed in half of the studies [33,34,35,40,42,43,44,45,50,52]. In four studies, patients enrolled in a multicomponent program with aerobic exercise, strength training, and psychological counseling [35,40,46,47], also adding nutritional counselling to one of them [47]. In one of the studies [35], aerobic exercise was delivered via virtual reality.

One study [36] combined aerobic and IMT, and two studies combined IMT with aerobic and strength training [43,52]. Study 43 also including lung expansion therapy. One study [37] compared IMT and usual care. RMT was combined in a complex program with aerobic and strength training in one study [42]. Two studies by the same first author [41,48] compare IMT and RMT with the same techniques, using a sham device as control [30] and combined aerobic and RMT with the same procedure but using a sham device for RMT [47]. Breathing exercises were performed in addition to each RCT protocol in six studies [35,38,39,45,47,52]. The intervention in one study [39] consisted of a wellbeing program comprising singing, psychological counselling, and breathing exercises.

Regarding how PR was administered, half of the studies included telerehabilitation programs, while the remaining were administered face-to-face. Three studies compared PR via telerehabilitation with an in-hospital PR program [35,46,47] or standard care [33,34,37,38,39,40,42,45]. Table 3 and Table 4 summarize the information from the included studies, indicating the first author, publication year, country, sample size and characteristics, intervention components, control group elements, outcomes, and statistically significant intragroup and intergroup results. The sample size presented in Table 3 reflects a complete-case analysis, comprising only those participants who completed the final assessment. This excludes individuals lost to follow-up due to intercurrent medical conditions, socio-professional commitments, logistical or technological impediments, and dissatisfaction with group allocation.

### 3.5. Outcomes

Detailed information including outcomes related to exercise capacity, pulmonary function, respiratory muscle function, peripheral muscle strength, quality of life, dyspnea, fatigue, anxiety and depression were extracted. High variety was identified, with different scales used to measure the same outcome.

Regarding exercise capacity, the 6-minute walk test (6MWT) and the cardiopulmonary exercise test (CPET) were the most used test to assess it, and to a lesser extent, the Chester step test, Ruffier test, and incremental shuttle walk test (ISWT). Patients’ qualities of life were assessed using different scales, but the 36-item short-form health survey (SF-36) and the 12-item short-form health survey (SF-12) were the most widely used, followed by the 5-dimension 5-level EuroQol questionnaire (EQ-5D-5L). Other studies used the St George’s Respiratory Questionnaire (SGRQ), the World Health Organization Quality of Life questionnaire (WHOQOL-BREF), the questionnaire for the quality of life in pulmonary diseases (K-BILD), and the patient-reported outcome measurement information system (PROMIS) preference score (PROPr).

Pulmonary function was evaluated by spirometry and body–plethysmography, and the following parameters were monitored: forced expiratory volume in the first second (FEV1), vital capacity (FVC), FEV1/FVC, peak expiratory flow (PEF), peak inspiratory flow (PIF), and diffusing capacity of carbon monoxide (TLCO). Respiratory muscle function was evaluated by maximal inspiratory pressure (MIP), maximal expiratory pressure (MEP), or sustained maximal inspiratory pressure (SMIP).

The modified Medical Research Council dyspnea scale (mMRC scale) was the most commonly used test for the assessment of dyspnea, followed by 12-item Dyspnea Questionnaire (Dyspnoea-12), Baseline Dyspnea Index (BDI), and Transitional Dyspnea Index (TDI). Fatigue had the most heterogeneous assessment, measured with many tools, such as the Fatigue Severity Scale (FSS), the 11-item Chalder Fatigue Scale (CFQ-11), the Modified Impact of Fatigue Scale (MFIS), the Multidimensional Fatigue Inventory (MFI) and Functional Assessment of Chronic Illness Therapy-Fatigue (FACIT-FS).

Anxiety and depression were generally rated using the Hospital Anxiety and Depression Scale (HADS) or by associating the Generalized Anxiety Disorder-7 (GAD-7) scale and the Patient Health Questionnaire-9 (PHQ-9), with the exception of one study which used the Beck Anxiety Inventory (BAI), and Beck Depression Inventory (BDI). Peripheral muscle strength was quantified by hand dynamometry, 30 s/1 min/five consecutive sit-to-stand test (30/1STS/5-STS), timed-up-and-go test (TUG), maximal voluntary contraction of the quadriceps (QMVC), and squats.

### 3.6. Effectiveness of Pulmonary Rehabilitation

#### 3.6.1. Exercise Capacity

The included studies were synthesized according to the predefined clinical outcomes. Exercise capacity was the most frequently evaluated outcome, followed by pulmonary function, respiratory muscle function, peripheral muscle strength, the quality of life, dyspnea, fatigue, anxiety, and depression. Considerable heterogeneity was observed regarding rehabilitation protocols, intervention duration, comparator groups, and outcome measures.

Nine studies [35,38,42,43,45,46,47,49,52] evaluated exercise capacity using the 6-minute walk test (6MWT). In the study by Elyazed et al. [42], despite a very short initial distance (baseline) (around 270 m), the telerehabilitation group achieved a spectacular improvement of +169 m (*p* < 0.001), compared to the +73 m improvement in the control group (*p* < 0.001), with the intergroup difference being extremely significant (*p* < 0.001). Li et al. [45] reported an adjusted difference of +65.45 m in favor of telerehabilitation after 6 weeks (*p* < 0.001). In the study by Okan et al. [38], the intervention group’s 6MWD significantly increased by 54.27 m (*p* < 0.001), while the control group’s increased by only 4.69 m, the intergroup difference being considered significantly larger in favor of the intervention (*p* < 0.001). Dos Santos et al. [43] reported the largest absolute improvement in 6MWD in the intervention group (+199.29 m) and the largest adjusted difference between groups (+100.46 m, *p* = 0.002).

Regarding the studies comparing PR with standard care, in three studies ([38,43,52]) pulmonary rehabilitation proved superior in improving 6MWD. Besnier et al. [52] reported an improvement of 6MWT by +47.7 m (*p* = 0.018) in the rehabilitation group, whereas the control group decreased by 14.2 m, resulting in a significant intergroup difference (*p* = 0.010). In the two studies [46,47] that compared tele-PR and in-hospital PR, Vallier et al. [46] identified significant intergroup improvement for both groups (*p* < 0.001), similar to the results of Arora et al. [47] showing significant improvement in both tele-PR (*p* < 0.01) and in-hospital rehabilitation (*p* = 0.03) groups. Rutkowski et al. [35] reported no significant difference between the group that added VR to in-hospital PR and the in-hospital PR, with both groups showing improvements (VR: +56.9 m, *p* < 0.001; Control: +39.2 m, *p* = 0.006) that exceeded the minimum detectable change (MDC).

The cardiopulmonary exercise test (CPET), Chester step test and ISWT were used to evaluate the exercise capacity in seven studies [33,34,37,44,48,51,52]. All three studies [44,51,52] that compared PR intervention with standard care demonstrated a statistically significant increase in VO_2_max in favor of the intervention. The study by Daynes et al. [34] demonstrated significant intragroup improvement in the study groups (in-hospital PR, *p* < 0.001 and tele-PR, *p* < 0.01) and the statistically significant superiority of both study groups compared to usual care (*p* < 0.05). In the study by Jimeno-Alamazan et al. [44], the intervention group showed a VO_2_max improvement of 2.1 mL/kg/min, with an intergroup difference of *p* = 0.035. Besnier et al. [52] reported a statistically significant effort capacity increase in the rehabilitation group of 2.73 mL/kg/min (*p* < 0.001), with favorable intergroup difference (*p* = 0.003) in favor of the rehabilitation group, and also a significant decrease in the ventilatory efficiency (VE/VCO_2_) from 30.04 + 7.72 at baseline to 27.47 + 3.94 after rehabilitation, with a statistically significant difference in favor of the intervention group (*p* = 0.032).

The most significant absolute improvement in metabolic parameters was reported by Bai et al. [51], with the predicted VO_2_ increasing in the HIIT/MIIT group by 4.65 mL/kg/min and 17%, *p* < 0.05, with significant intergroup difference in favor of the rehabilitation group (*p* < 0.001). In the study by del Corral et al. [48], which compared the benefits of AE + RMT with AE + RMTsham, the VO_2_max (mL/kg/min) statistically increased significantly intragroup in both groups, but the intergroup difference was not significant (*p* = 0.532). McNarry et al. [37] demonstrated similar results, with a significant VO_2_max intragroup increase in the intervention group (p<0,05), but no statistical difference between groups. Longobardi et al. [33] found no significant differences in the VO2max (mL/kg/min) increase between the group that followed a home rehabilitation protocol and the control group that received advice on a healthy lifestyle.

#### 3.6.2. Pulmonary Function

Eleven studies [33,36,38,41,44,45,46,47,48,51,52] assessed pulmonary function, with varying results. Longobardi et al. [33], Jimeno-Alamazan et al. [44], Bai et al. [51], and Besnier et al. [52] reported no statistically significant intragroup or intergroup improvements in the parameters. TLCO significantly increased in the intervention group in the study by del Corral et al. [48] (*p* = 0.035), and in both groups in the study by Vallier et al. [46] (*p* = 0.009), with no significant intergroup differences. Del Corral et al. [48] reported a significant intragroup increase in PEF in the intervention group (*p* = 0.004) and a statistically significant intergroup difference in favor of the intervention (*p* < 0.005). PEF also significantlly increased in the I1 group of the del Corral et al. [41] study and also between group comparing groups I1 vs. C1, I2 vs. I1 and C2 vs. I1. In the study by Okan et al. [38], FEV1, FVC, and MVV significantly increased in the intervention group (*p* < 0.001), but only the MVV increased intergroup with statistical significance (*p* < 0.001). Sanchez-Milla et al. [36] reported a significant increase in FVC and FEV1/FVC both intragroup in the intervention group (*p* < 0.003, respectively, *p* < 0.001) and intergroup (*p* < 0.001). The MVV significantly increased intergroup in the study by Li et al. [45] (*p* = 0.005), while the FVC significantly increased (*p* = 0.004) in the control group of the study by Arora et al. [47] and in both groups of Vallier et al. [46] (*p* = 0.011).

#### 3.6.3. Respiratory Muscle Function

Maximal inspiratory pressure (MIP) was evaluated in five studies [36,37,41,48,49], and maximal expiratory pressure (MEP) in two studies [41,48]. Sanchez-Milla et al. [36], McNarry et al. [37], and Nagy et al. [49] reported a significant intragroup MIP increase in the intervention group (*p* < 0.001, respectively, *p* < 0.05 in study 49). Intergroup MIP also significantly increased, with a significance of *p* < 0.001 (36), *p* < 0.01 [37], and *p* < 0.05 [49].

In the first study by del Corral et al. [41], the MIP significantly increased in both intervention groups IMT and RMT (*p* < 0.001), and both control groups RMT sham and IMT sham (*p* < 0.006 and *p* = 0.04). The MEP significantly increased intragroup in both intervention groups (*p* < 0.001) and in both control groups (*p* < 0.001 RMTsham and *p* = 0.032 IMT sham group). Both MIP and MEP increased significantly intergroup between RMT and RMT sham (*p* < 0.01). Intergroup significant differences regarding MEP were also observed between IMT and RMT (*p* < 0.01), in favor of IMT and between IMTsham and RMT (*p* < 0.01), in favor of IMTsham. Intergroup significant differences were observed in MIP increase between IMT and both control groups (*p* < 0.05), in favor of IMT and between IMTsham and RMTsham (*p* < 0.01) in favor of IMTsham.

In the second study by del Corral et al. [48], MIP significantly increased intragroup in the intervention and control groups (*p* < 0.001 and *p* = 0.043), but MEP increased only in the intervention group (*p* < 0.001). The intergroup difference was significant in favor of the intervention (*p* = 0.004).

#### 3.6.4. Peripheral Muscle Strength

Peripheral muscle strength was assessed in eight studies [33,34,41,44,45,46,48,52]. Vallier et al. [46] compared in-hospital PR with tele-PR demonstrated significant intragroup changes in both groups, without significant intergroup differences. In two studies [33,45] that compared PR and a control group receiving standard care, no significant intragroup differences were found, but only intergroup differences in favor of the intervention group were found (33–30STS, *p* = 0.048; 45-Sq *p* < 0.001). Jimeno-Alamazan et al [44] compared PR and a control group receiving educational counselling and fond significant intragrup changes in both groups, with pulmonary rehabilitation proving statistically superior (44-STS5 *p* = 0.009, BP 50%1RM *p* = 0.012, HSQ 50%1RM *p* = 0.032). Daynes et al. [34] reported significand handgrip improvement in the tele-PR and control groups, with significant difference only between the in-hospital PR group and control group. In the same study, the QMVC was improved in both intervention groups, but significant improvements were demonstrated only between the tele-PR and control group, in favor of tele-PR. Del Corral et al. [41] reported that both intervention groups showed significant improvements in the 1STS test (*p* < 0.001), and all groups showed significant increases in manual dynamometry (*p* < 0.05). 1STS improvement was significant between the intervention groups and their control counterparts (*p* < 0.01, respectively, *p* < 0.05). In the second del Corral et al. [48] study, the 1STS significantly increased in both groups (*p* < 0.01), without significant intergroup differences. Comparing PR and no intervention, Besnier et al. [52] reported a significant time decrease in the intervention group for TUG usual speed (*p* = 0.004) and TUG fast speed (*p* = 0.008), with a significant difference in TUG usual speed between groups in favor of the intervention (*p* = 0.031).

#### 3.6.5. Quality of Life

Multiple tools and questionnaires were used to evaluate the impact of the interventions on the quality of life in sixteen studies [33,34,35,37,38,39,40,41,42,44,45,46,48,50,51,52]. Eight studies [33,39,42,44,45,50,51,52] evaluated the impact of post-COVID-19 syndrome using the SF-36 or SF-12 questionnaires. Jimeno-Alamazan et al. [44], Li et al. [45], and Romanet et al. [50] reported a statistically significant increase in the physical component of the SF-12 score in favor of the intervention group (PR program), with no differences in the mental component. The SF-36 score was significantly improved in favor of the intervention group in the studies by Besnier et al. [52] (physical and mental health domains, *p* < 0.05), Philip et al. [39] (mental component, *p* = 0.047), and Longobardi et al. [33] (physical component *p* < 0.001). The total SF-36 score had the greatest reported significant increase (+40 points, *p* < 0.001) in the Elyazed et al. [42] study. Bai et al. [51] reported no significant intergroup differences.

Four studies [34,40,41,48] assessed the quality of life using the EQ-5D-5L questionnaire. The del Corral et al. [41] study demonstrated significant improvements in favor of the intervention group (*p* < 0.001), while the second del Corral et al. [48] study only demonstrated significant intragroup improvements, *p* < 0.001. The study by McGregor et al. [40] assessed quality of life as the primary outcome using the PROPr score and EQ-5D-5L, both demonstrating significant intergroup improvement (*p* = 0.02 for PROPr and *p* < 0.04 for EQ-5D-5L VAS score) in favor of the intervention. No significant intragroup or intergroup improvements were reported in the Daynes et al. [34] study.

Four studies uniquely quantified the impact of the syndrome on the quality of life using the K-BILD questionnaire [37], VQ-11 questionnaire [46], SGRQ questionnaire [38], and WHOQOL-BREF questionnaire [35]. In the study by Vallier et al. [46], although no significant intergroup benefits were demonstrated, both groups showed statistically significant improvements in the quality of life, *p* < 0.001. The total K-BILD score and scores per domains significantly increased intragroup in McNarry et al.’s [37] study. Although the SGRQ score significantly decreased in both studied groups (*p* < 0.001 I and *p* < 0.007 C), in the Okan et al. [38] study, the intergroup difference in favor of the intervention was statistically significant (*p* < 0.001). The Rutkowski et al. [35] study reported no intergroup or intragroup significant difference.

#### 3.6.6. Anxiety and Depression

Anxiety and/or depression were rated in nine studies [33,34,35,39,40,41,44,48,51] with mixed results. No significant improvements were identified neither intragroup nor intergroup in the study by Daynes et al. [34]. In the studies by McGregor et al. [40] and Jimeno-Alamazan et al. [44], only depression decreased intergroup in favor of I (*p* = 0.013, respectively, *p* < 0.05), and in the study by Bai et al. [51], depression significantly improved (*p* < 0.05) in I group. Both affective imbalances decreased intragroup in both groups (*p* < 0.001) of the study conducted by Rutkowski et al. [35] and del Corral et al [48], and in the intervention and C1 groups of the study by del Corral et al. (*p* < 0.001) [41]. Anxiety was significantly diminished intergroup in the Philip et al. [39] study.

#### 3.6.7. Dyspnea

Sixteen studies [34,35,36,37,38,39,40,42,43,44,45,46,47,49,50,52] assessed dyspnea as an outcome. The majority of the studies [35,37,38,39,43,44,45,49,50,52] state significant intergroup differences regarding dyspnea improvement in favor of the intervention. The studies by Sanchez-Milla et al. [36] and Elyazed et al. [42] identified significant intragroup (*p* < 0.001) and intergroup (*p* < 0.001, respectively, *p* < 0.008) differences. Furthermore, virtual reality interventions demonstrated a significant improvement in dyspnea in the Rutkowski et al. [35] study (*p* = 0.033) and tele-PR and in-hospital PR demonstrate the same significant impact on dyspnea in the study conducted by Vallier et al. [46]. Daynes et al. [34] and Arora et al. [47] found no statistically significant intergroup or intragroup difference between in-hospital pulmonary rehabilitation vs. standard care or tele-PR versus standard care. McGregor et al. [40] study reported similar results, with no significant difference intragroup or between groups.

#### 3.6.8. Fatigue

Regarding fatigue, the results from seven studies [33,34,42,43,44,46,49] were more consistent. Fatigue was significantly reduced in the in-hospital rehabilitation group in the studies conducted by Daynes et al. [34] and Vallier et al. [46], but intergroup difference was demonstrated only in the study that compared in-hospital PR and tele-PR [46] in favor of the in-hospital PR group (*p* = 0.016 MFI total score). Two studies [42,49] showed a significant fatigue reduction in both compared groups and also in the intergroup comparison (*p* < 0.001 in both RCTs). Two studies compared cardiopulmonary rehabilitation [43], respectively, pulmonary rehabilitation [44] with educational counselling. While the study conducted by dos Santos et al. [43] demonstrated a significant fatigue reduction only in the intervention group (*p* = 0.028), the Jimeno-Alamazan et al. [44] study demonstrated significant fatigue improvement in both intervention group (*p* = 0.01 for CFQ-11, *p* = 0.02 for FSS) and intergroup in favor of PR (*p* = 0.007 for CFQ-11, *p* = 0.024 for FSS). The study by Longobardi et al. [33] did not identify significant changes, although it observed a trend toward a decrease in the proportion of fatigued patients in the exercise group.

#### 3.6.9. In-Hospital Rehabilitation vs. Telerehabilitation

Two studies, conducted by Vallier et al. [46] and Arora et al. [47], compared in-hospital PR interventions with telerehabilitation. The Vallier et al. [46] study demonstrated significant statistical increase in both groups regarding the outcomes 6MWD, pulmonary function, dyspnea, quality of life and peripheral muscle force, with no statistically significant intergroup difference. The only outcome that demonstrated the superiority of inpatient PR over home-based rehabilitation was in the domain of fatigue, assessed by the MFI total score (*p* = 0.016), with a significant decrease in the in-hospital group (*p* < 0.001). The Arora et al. [47] study demonstrated similar statistically significant intergroup improvement regarding 6MWD in both groups (*p* < 0.03 for in-hospital group and *p* < 0.01 for tele-PR group) and FVC increase only in the in-hospital rehabilitation group (*p* < 0.04), without differences between groups.

#### 3.6.10. Telerehabilitation vs. Standard Care/Educational Counselling

Six studies [33,34,37,38,42,45] compared telerehabilitation and standard care/educational counselling. The studies conducted by Daynes et al. [34], Okan et al. [38], Elyazed et al. [42], and Li et al. [45] demonstrated the superiority of telerehabilitation for improving the exercise capacity (34 *p* = 0.047, 38 *p* < 0.001, 42 *p* < 0.001, 45 *p* < 0.001). In the McNarry et al. [37] study, although there was a significant VO_2_max improvement in the intervention group, there was no significant difference between groups. Longobardi et al. [33] found no significant difference intragroup or between groups.

The studies that assessed dyspnea also reported significant improvement in favor of the tele-PR group [38,42,45]. The quality of life was also significantly improved in three studies [38,42,45] in favor of tele-PR (38, *p* < 0.001, 42 *p* < 0.001, 45 *p* = 0.004). No significant difference was reported on the dyspnea perception in the Daynes et al. [34] study. Pulmonary function was significantly improved in the tele-PR group compared to the educational counselling group in the study conducted by Okan et al. [38], *p* < 0.001. Studies 38 and 45 demonstrated the intervention was superior to the control for MVV, suggesting that the exercises effectively targeted respiratory muscle performance but did not modify the underlying lung structure or basic pulmonary volumes. The study conducted by McNarry et al. [37] reported a significant intergroup improvement in favor of tele-PR in the MIP outcome (*p* < 0.05), and Li et al. [45] reported the same results, but concerning the lower-limb muscle strength (*p* < 0.001).

## 4. Discussion

Considering the results obtained from the various included RCTs, it is evident that pulmonary rehabilitation is the key therapeutic approach of post-COVID19 syndrome, with sustained benefits regarding exercise capacity, respiratory and peripheral muscle function, dyspnea, and the quality of life.

The most effective rehabilitation protocols for exercise capacity improvement were the most sophisticated ones, including aerobic, strength training, and other rehabilitation means [42,43,44,51,52]. Dyspnea was reduced the most in the studies in which the intervention consists of at least 2 session/week of aerobic training [44,45]. Regarding comprehensive respiratory muscle force (both MIP and MEP), the study by del Corral et al. [41] including an 8-week home-based RMT protocol is the superior choice for both MIP and MEP. IMT is the superior intervention on MIP increase compared to IMTsham or RMTsham. Fatigue diminution was consistently reduced in the majority of the studies [34,42,43,44,46,49].

The review found robust effectiveness of pulmonary rehabilitation for the lower-limb muscle strength. The majority of the studies [33,41,44,46] reported significant improvements in sit-to-stand (STS) or squat tests. For instance, the TERECO program [45] achieved an improvement of over 20 s in the static squat test, a treatment effect that was sustained for 7 months (estimated at 20.12 s post-treatment and 22.23 s at follow up). There are mixed results for the upper-limb muscle force, handgrip strength showing less consistent improvements. While Study PHOSP-R [23] reported a significant increase in handgrip strength specifically within the face-to-face rehabilitation group (+2.06 kg) compared to usual care, other research, such as the del Corral et al. studies [41,48], found no significant intergroup differences for this particular variable.

Despite the absence of a standardized protocol for training patients with COVID-19, exercising was consistently incorporated in almost every study, emphasizing its significance in managing the post-COVID-19 condition. The inclusion of exercise highlights its role as a core component in the therapeutic approach for patients undergoing rehabilitation from COVID-19.

A key contribution of this work is the nuanced interpretation of heterogeneous data related to spirometry. The heterogeneous spirometric results are explained by the fact that pulmonary rehabilitation protocols target muscular and metabolic conditioning rather than lung structure, with many patients already presenting with normal baseline values that limit the potential for statistical improvement. It explicitly emphasizes that functional improvements are primarily due to physical reconditioning and muscle training (including respiratory muscles), rather than major changes in intrinsic pulmonary pathology (reflected in FEV1/FVC). This critical distinction suggests a deeper understanding of the mechanism of action of PR in post-COVID-19 syndrome.

### 4.1. Study Strengths

Given that the study was conducted between January and February 2026, it can be considered an up-to-date review of the specialized literature, serving as a current reference on the subject.

Firstly, the article is based exclusively on randomized clinical trials (RCTs) and defines an exhaustive set of primary and secondary evaluation parameters that must have been monitored in the included studies. This methodological rigor ensures a high-quality evidence base, reducing bias risk and increasing the reliability of conclusions. The broad range of parameters (exercise capacity, QoL, respiratory muscle function, dyspnea, fatigue, peripheral muscle strength, anxiety, and depression) demonstrates a highly comprehensive approach.

Secondly, the study is not limited to a few standard indicators but analyzes a multitude of specific instruments for each domain (e.g., 6MWT, CPET for effort capacity; SF-36, EQ-5D-5L, K-BILD, SGRQ, WHOQOL-BREF for QoL). This detailed presentation of measurement instruments allows for a more nuanced understanding of intervention effects and highlights the exhaustiveness of the analysis.

Thirdly, this review acknowledges the importance of tele-PR, and focuses on directly comparing the efficacy between tele-PR and in-hospital PR or standard care in half of the RCTs included. This shows that their efficacy is similar in terms of exercise capacity, confirming that tele-PR is a viable alternative for increasing accessibility. This direct comparative approach offers practical clinical guidance. The article not only consolidates general evidence but also makes specific and clinically relevant comparisons. These comparisons provide practical guidance on choosing the most appropriate intervention modality, depending on resources and patient context:

Telerehabilitation vs. standard care: Elyazed et al. [42] demonstrates a significant improvement of +169 m in 6MWT in the telerehabilitation group compared to +72.9 m in the control group.

Telerehabilitation vs. in-hospital rehabilitation: Vallier et al. [46] and Arora et al. [47] show similar efficacy between the two, a crucial conclusion for adapting medical services.

Fourthly, this review openly acknowledges where results are heterogeneous (e.g., pulmonary function, mental components of QoL, anxiety, and depression) and suggests the need for personalized approaches. This scientific honesty adds credibility and indicates clear directions for future research and clinical adaptation.

### 4.2. Methodological Shortcomings and Study Limitations

It is important to acknowledge certain methodological shortcomings of the study. Firstly, only half of the included RCTs were rated with a low risk of bias, an aspect that can affect the validity of the conclusions. Secondly, some of the RCTs include a low sample size, and the outcomes were reported short-term, at the end of the intervention, only five studies [33,40,42,45,50] reporting long-term effects, at minimum of three months from the intervention. The low sample size limits the statistical power, and the short-term reporting of the effects limits the understanding of long-term benefits.

Thirdly, the intervention/control protocols are variable, from complex interventions combining aerobic, strength training, and respiratory muscle training/breathing techniques [35,42,43,45,47,52], to more simplistic ones including only IMT/RMT [37,41] or aerobic training [44,51]. This heterogeneity can limit the ability to draw definitive conclusions.

Fourthly, only studies published in English were included, grey literature was not searched, and no quantitative meta-analysis or GRADE assessment was performed because of substantial clinical heterogeneity, limiting the statistical power, and preventing a more clear estimation of the intervention’s effects.

Several limitations should be considered when interpreting the available evidence. Most rehabilitation protocols varied substantially with respect to duration, intensity, and intervention components, and different outcome measures were used across studies. These factors limited direct comparisons between trials and precluded quantitative pooling of results. Because a quantitative meta-analysis was not performed, no pooled effect estimates or measures of statistical heterogeneity are reported. The certainty of evidence was not formally evaluated using the GRADE approach because of the considerable clinical heterogeneity among the included randomized controlled trials and the absence of quantitative synthesis.

### 4.3. Comparison with the Existing Literature

Other systematic and narrative reviews are in line with the results obtained in this study. Four reviews also support the significant improvement in the effort capacity, measured by 6MWD [53,54,55,56] or VO2max [56], in the group that received pulmonary rehabilitation as intervention, confirming the improvement in exercise capacity as a consistent result of pulmonary rehabilitation. The same four reviews present heterogeneous or nuanced results regarding spirometric pulmonary function, but two of them [53,56] highlight the remarkable efficacy of respiratory muscle training benefits for respiratory muscle strength, with a significant increase in MIP, similar to the findings in this review.

Regarding dyspnea easing and significant improvement in the quality of life, the results of this study are similar to other reviews [53,55,57,58,59], but the results on fatigue are heterogeneous, with a significant reduction [57], similar to the results of this review, or no effect [60]. All studies support significant improvements in peripheral muscle force, similar to the results from other reviews [61,62,63]. Regarding anxiety and depression, other reviews had similar heterogeneous results, some stating benefits on depression [60] and others a positive impact on anxiety [58].

Regarding telerehabilitation, the review conducted by Sakai et al. [54] stated that tele-rehabilitation is an effective tool, showing its superiority (web-based, app-based) over the lack of treatment for ameliorating dyspnea, muscle strength, and walking capacity. The results were similar to those reported from this review, showing the superiority of tele-PR over usual care/educational counselling in for ameliorating exercise capacity, dyspnea, quality of life, pulmonary function, pulmonary and peripheral muscle strength,

The review conducted by Fernandez-Lazaro et al. [56] mentioned that telemedicine programs demonstrated similar benefits to traditional interventions in reducing dyspnea, improving functional capacity, and QoL, results that are similar to the results of this review. Another review, conducted by da Salva Vieira et al. [64], concluded that exercise programs delivered via telerehabilitation may improve functional capacity, lower-limb performance, dyspnea and the physical component of the quality of life compared with no rehabilitation in patients with post-COVID-19 condition. The conclusion on functional capacity is similar to the conclusion on this review, with significant improvement in the exercise capacity in the RCTs that compared tele-PR and standard care/educational counselling [34,37,38,42,45] in favor of tele-PR.

Future randomized controlled trials should adopt standardized pulmonary rehabilitation protocols and harmonized outcome measures to facilitate direct comparisons and future meta-analyses. Further research is also required to determine the optimal duration, intensity, and delivery modality of pulmonary rehabilitation, particularly regarding telerehabilitation, and to identify patient subgroups that derive the greatest benefit from rehabilitation interventions.

## 5. Conclusions

Overall, the consolidated data from this review underscore the pivotal role of pulmonary rehabilitation in the complex management of post-COVID-19 syndrome.

The capacity of pulmonary rehabilitation to improve exercise tolerance, respiratory and peripheral muscle function, quality of life, and reduce dyspnea and fatigue is well-founded.

Telerehabilitation represents a viable and efficient intervention modality, demonstrating comparable efficacy to in-hospital pulmonary rehabilitation and expanding access to these essential services.

The heterogeneity of the results for certain parameters, such as pulmonary function (FEV1, FVC, FEV1/FVC), anxiety, and depression suggests that the mechanisms of action are predominantly linked to physical reconditioning and muscle training, rather than major structural pulmonary changes.

Continued research is essential to elucidate optimal protocols, intervention durations, and patient groups that benefit most from certain types of exercises.

The integration and validation of multidisciplinary interventions addressing the psychological and systemic aspects of the syndrome remain a priority to maximize patient benefits.

### Points for Clinical Practice

The importance of the early implementation of pulmonary rehabilitation in patients with post-COVID-19 syndrome is well established.

These interventions complement conventional treatment in a synergistic manner. Where appropriate, telerehabilitation may succesfully replace hospital-based rehabilitation programs and should be recommended to all patients who are able to adhere to the prescribed therapeutic regimen.

## Figures and Tables

**Table 1:** Symptomatology of post-COVID-19 syndrome.

Injury	Symptoms
Respiratory	Cough, dyspnea, chest pain
Cardiovascular	Chest pain, palpitations, myocarditis, orthostatic postural tachycardia, ischemic heart disease
Nervous	Fatigue, brain fog, amnesia, paresthesia
Psycho-emotional	Anxiety, depression, mood changes, sleep disorders, PTSD
Musculoskeletal	Myalgia, arthralgia
Gastrointestinal	Nausea, diarrhea/constipation, abdominal pain, decreased appetite

**Table 3:** Characteristics of the sample in the selected studies.

Author (Year)	Country	COVID-19 Severity	Sample Size T, I/C	Sample Characteristics
[33] Longobardi et al. (2023)	Brazil	Severe/critical	50 25/25	Age (mean ± SD) = 61 ± 7.4 F = 23 (50%)
[34] Daynes et al. (2024)	UK	Non-severe/Severe/critical	181 I1 56/I2 63/C 62	Age (mean ± SD) = 56 ± 12 F = 82 (45,3%)
[35] Rutkowski et al. (2022)	Poland	A classification (mild/severe/critical) is not specified	32 18/14	Age (mean ± SD) = 57.8 ± 4.9 F = 22 (68.8%)
[36] Sanchez Milla et al. (2024)	Spain	Mild	200 100/100	Age (median) = 22 F = 101 (50.5%)
[37] McNarry et al. (2022)	UK	A classification (mild/severe/critical) is not specified	148 111/37	Age (mean ± SD) = 46.6 ± 12.2 F = 130 (87,8%)
[38] Okan et al. (2022)	Turkey	A classification (mild/severe/critical) is not specified	52 26/26	Age (mean ± SD) = 50.49 ± 12.85 F = 25 (48.1%)
[39] Philip et al. (2022)	UK	A classification (mild/severe/critical) is not specified	129 58/71	Age (median) = 49 F = 103 (79,8%)
[40] McGregor et al. (2024)	UK	A classification (mild/severe/critical) is not specified	485 237/248	Age (mean ± SD) = 56.1 ± 12.2 F = 252 (52%)
[41] del Corral et al. (2022)	Spain	A classification (mild/severe/critical) is not specified	88 I1 22/I2 22/C1 22/C2 22	Age (mean ± SD) = 46.42 ± 10.22 F = 63 (71.59%)
[42] Elyazed et al. (2024)	Egypt	Moderate/Severe	60 30/30	Age (mean ± SD) = 55.75 ± 6.9 F = 27 (45%)
[43] dos Santos et al. (2024)	Brazil	A classification (mild/severe/critical) is not specified	33 17/16	Age (mean ± SD) = 47.38 ± 12.8 F = 20 (60,6%)
[44] Jimeno-Alamazan et al. (2022)	Spain	Mild	39 19/20	Age (mean ± SD) = 45.3 ± 9.7 F = 29 (74.4%)
[45] Li et al. (2021)	China	Non-severe/Severe	112 52/60	Age (mean ± SD) = 50.6 + 10.98 F = 62 (52%)
[46] Vallier et al. (2023)	France	Mild/Moderate/Severe	17 9/8	Age (mean ± SD) = 54.8 ± 16 F = 5 (29.41%)
[47] Arora et al. (2025)	India	Mild/Moderate/Severe	54 27/27	Age (mean ± SD) = 50.8 ± 14.7 F = 29 (53,7%)
[48] del Corral et al. (2025)	Spain	A classification (mild/severe/critical) is not specified	64 32/32	Age (mean ± SD) = 50.3 ± 10.5 F = 41 (64.1%)
[49] Nagy et al. (2022)	Egypt	A classification (mild/severe/critical) is not specified	52 26/26	Age (mean ± SD) = 39.85 ± 3.45 F = 0
[50] Romanet et al. (2023)	France	A classification (mild/severe/critical) is not specified	60 27/33	Age (mean ± SD) = 58 + 12.11 F = 23 (38,3%)
[51] Bai et al. (2024)	China	A classification (mild/severe/critical) is not specified	24 12/12	Age (mean ± SD) = 45.23 ± 15.11 F = 14 (58.3%)
[52] Besnier et al (2022)	Canada	A classification (mild/severe/critical) is not specified	35 18/17	Age (mean ± SD) = 53.18 ± 11.71 F = 24 (52,5%)

Abbreviations: S = sample size, T = total number of participants, I = intervention group, C = control group, F = % of females, SD = standard deviation, UK = United Kingdom of Great Britain and Northern Ireland.

**Table 4:** Description of intervention and control, and intragroup and intergroup statistically significant results (*p* < 0.05).

	Intervention (Type, Duration, Components)	Control	Measured Parameters	Intragroup res (*p* < 0.05)	Intergroup res (*p* < 0.05)
[33] **Longobardi et al. (2023)**	TELE-PR 3 sessions/week, 60–80 min/session, 16 weeks aerobic (walking, jogging): from short session (10 min/day) to one session of 50 min/day strength training 6 ex, 3–5 sets, 8–15 reps. Progressive training intensity based on RPE 1 supervised video session/week	Standard care (healthy lifestyle advices, diet, physical activity)	Exercise capacity:		
			CPET VO_2_max (L/min)	↔	↔
			VO_2_max (mL/kg/min) VO at VT1	↔ ↔	↔ ↔
			VO at VT2 VE/VCO_2_	↔ ↔	↔ ↔
			HRQOL:		
			SF-36	↑ PCS + PF, GH, VT	↑ PCS + PF, GH, VT
			Pulmonary function:		
			FEV1, FVC, FEV1/FVC, PIF, PEF	↔ ↔	↔ ↔
			PMS:		
			30STS	↔	↑
			Handgrip	↔	↔
			TUG	↔	↔
			Fatigue: FSS	↔	↔
			Anxiety and depression:		
			BAI	↔	↔
			BDI*	↔	↔
[34] **Daynes et al. (2024)**	I1. PR 3 sessions/week in hospital, 90–120 min/session, 8 weeks aerobic, walking strength training auto management strategies educational counselling I2. Tele-PR on your COVID rehabilitation Platform 4 phases, 1 phase = 2 weeks aerobic, walking strength training	Standard care, depending on symptoms	Exercise capacity:		
			ISWT (VO_2_max)	↑ I1,I2	↑ I1 vs. C, ↑ I2 vs. C
			HRQOL:		
			EQ-5D-5L	↔	↔
			Anxiety and depression:		
			GAD-7	↔	↔
			PHQ-9	↔	↔
			Dyspnea:		
			Dyspnoea-12	↔	↔
			mMRC	↔	↔
			Fatigue:		
			FACIT-FS	↑ I1,C	↔
			PMS:		
			Handgrip	↑ I2,C	↑ I1 vs. C
			QMVC	↑ I1,I2	↑ I2 vs. C
[35] **Rutkowski et al. (2022)**	PR VR-assisted 3 weeks aerobic (cycloergometer) on a sunny island, with heart rate limits vary by the rehabilitation models included (model A-80% of SHR, model B-70% SHR, model C-60% SHR, model D-20–30% HRR) strength training VR relaxing techniques–therapeutical virtual garden breathing exercises	IN-HOSPITAL 5 sessions/week, 3 weeks aerobic (cycloergometer) with heart rate limits vary by the rehabilitation models included (model A-80% of SHR, model B-70% SHR, model C-60% SHR, model D-20–30% HRR) strength training mindfulness techniques breathing exercises	Exercise capacity:		
			6MWT	↑	↔
			HRQOL:		
			WHOQOL-BREF	↔	↔
			Anxiety and depression:		
			HADS-A/D	↓ I,C	↔
			Dyspnea:		
			mMRC	↓	↓
[36] **Sanchez Milla et al. (2024)**	PR + neurological rehab 4 weeks IMT: every day, 5 min/day, with Powerbreathe device aerobic (walking): 6 days/week, 1 session = 40 min/day, intensity of 60–75% MHR and 50–60% MHR olfactory and gustatory rehabilitation	No treatment	Pulmonary function:		
			FEV1, FVC, FEV1/FVC	↑ FVC, ↑ FEV1/FVC	↑ FVC ↑ FEV1/FVC
			Respiratory muscles function: MIP	↑ MIP I	↑
			Dyspnea:		
			mMRC	↓	↓
			Borg scale	↓	↓
			Neuro: SSTQ	↑	↑
[37] **McNarry et al. (2022)**	TELE-PR IMT 3 unsupervised sessions/week, 20 min/session, 8 weeks IMT with PrO2Fit device, 1 session = 6 cycles of 6 breaths, in non-consecutive days before each session MIP is measured, to maintain IMT pressure > 80% MIP during training	Standard care, depending on symptoms	Exercise capacity:		
			Chester step test		
			VO_2_max (mL/kg/min)	↑ I	↔
			Respiratory muscles function:		
			MIP, SMIP	↑ MIP I ↑ SMIP I	↑ MIP ↑ SMIP
			HRQOL:		
			K-BILD	↑ total score + breathless and activities domain, psychological domain	↔
			Dyspnea:		
			BDI	↔	↔
			TDI	↑	↑
[38] **Okan et al. (2022)**	TELE-PR 5 weeks aerobic (walking) 20–30 min/day, 5 days/weeks, intensity <3 RPE breathing exercises 3 sessions × 10 ex, daily, 2 h after meal	Educational counselling: brochure with breathing exercises	Exercise capacity:		
			6MWT	↑ 6MWD I	↑↑ 6MWD
			HRQOL: SGRQ	↓ I,C	↓
			Pulmonary function:		
			FEV1, FEV1/FVC, MVV	↑ FEV1, ↑ FVC ↑ MVV	↑ FEV1, ↑ FVC ↑ MVV
			Dyspnea:mMRC	↓ I,C	↓
[39] **Philip et al. (2022)**	PR ENO Breathe protocol, 6 weeks Description: online program that integrates singing techniques to optimize breathing control and reduce anxiety 1 online group session/week: singing lullabies breathing exercises, vocal training, postural training anxiety and depression auto management techniques	Standard care, depending on symptoms	HRQOL: SF-36	↔	↑ MCH score
			Dyspnea: VAS	↔	↓
			Anxiety: GAD-7	↔	↓
			Dyspnea:		
			Dyspnoea-12	↔	↔
			VAS	↔	↓ VAS breathless running
[40] **McGregor et al. (2024)**	TELE-PR 8 weeks 2–3 sessions/week, 30 min/session, group supervised training aerobic strength training balance and coordination improvement 6 sessions/week, 1 h/session, group-supervised psychological training	1 consult of 30 min about post-COVID syndrome and its implications NHS COVID recovery brochure	HRQOL:		
			PROPr	↔	↑
			EQ-5D-5L	↔	↑ VAS score
			Anxiety and depression: HADS-A/D	↔	↓ HADS-D
			Dyspnea: PROMIS	↔	↔
		
[41] **del Corral et al. (2022)**	PR 12 sessions/week, morning and evening, 20 min/session, 6days/week, 8 weeks I1 = RMT I2 = IMT Morning session—unsupervised Evening session—supervised via videoconference MIP/MEP: S1 + S5: I1 50% MIP, I2 20% MIP/MEP S2 + S6: 60% MIP/MEP S3 + S7: 70% MIP/MEP S4 + S8: 80%MIP/MEP	TELE-PR identical protocol like in I group C1 = RMT sham C2 = IMT sham C1, and C2 groups used a sham device RMT/IMT (0 cm H2O resistance)	Exercise capacity:		
			Ruffier test	↑ I1	↔
			Respiratory muscles function:		
			MIP MEP	↑ MIP I1, I2, C1, C2 ↑ MEP I1, I2, C1, C2	↑ MIP I2 vs. C1/C2 ↑ MIP I1 vs. C1 ↑ MIP C2 vs. I1 ↑ MEP I2 vs. C2 ↑ MEP I1 vs. C1 ↑ MEP C2 vs. I1
			HRQOL: EQ-5D-5L INDEX/VAS	↑ Index + VAS I1, I2, C2	↑ Index I1 vs. C1 ↑ VAS I1 vs. C1
			Pulmonary function:		
			FEV1, FVC, FEV1/FVC, PEF	↔ ↑ PEF I1	↔ ↑ PEF I2 vs. I1, I1 vs. C1, C2 vs. I1
			PMS:		
			Handgrip	↑ I1, I2, C1, C2	↔
			1STS	↑ I1, I2	↑ I1 vs. C1 ↑ I2 vs. C2 ↑ I2 vs. C1 ↑ C2 vs. I1
			Anxiety and depression:		
			HADS	↑ I1, I2, C1, C2	↔
			Dyspnea: VAS	↔	↔
[42] **Elyazed et al. (2024)**	TELE-PR 12 weeks aerobic (walking): 30 min-1 h/day, 1 session/day, 5 days/week RMT 10–15 min, 2 sessions/day, 5 days/week strength training (sup limbs + quadriceps) 3 sets × 10 reps, 2 sessions/week, 5–7 days/week diaphragmatic strengthening with 1–2 kg loads on the patients’ abdomen	Standard care, depending on symptoms: multivitamins, Antioxidants, hyperproteic diet	Exercise capacity:		
			6MWT	↑ I,C	↑
			HRQOL: SF-36	↑ I,C	↑
			Dyspnea: mMRC	↓ I,C	↓
			Fatigue: CFQ-11	↓ I,C	↓
[43] **dos Santos et al. (2024)**	CARDIOPULMONARY R 6 weeks IMT with Threshold device, 3 sets × 10 reps, with 1 min pause between steps, MIP 40% W1–3, MIP 50% W3–6 pulmonary expansion therapy EPAP and PEEP 5–20 cm H2O, 3 sets × 2 min, with 2 min pause between sets aerobic (treadmill) 20 de min/day, progressive intensity 60%, 70% and 80% in W1, W5, W6 strength training sup limbs, initially at 50% VO2max and progressive increase if RPE 4–6	Educational counselling lifestyle recommendations	Exercise capacity:		
			6MWT	↑	↑
			Dyspnea: mMRC	↓	↓
			Fatigue: MFIS	↓	↔
			Body composition: upper- and lower- limb muscle mass	↑ upper-limb muscle mass	↑ upper-limb muscle mass
[44] **Jimeno-Alamazan et al. (2022)**	PR 3 sessions/week, 8 weeks 1 session/week of continuous low intensity continuous training (65–70% HRR), 30–60 min 2 sessions/week: continuous moderate intensity-variable training (3–5 min of 65–70% HRR, then 2–3 min of 55–65% HRR), 30–60 min + strength training, 3 sets of 4 ex, 8 reps, 50% of 1RM intensity	Educational counselling: WHO Guidelines of rehabilitation and self-management after COVID-19	Exercise capacity:		
			CPET VO_2_max (mL/kg/min)	↑ I	↑
			HRQOL: SF-12	↑ PA domain	↑ PCS
			Pulmonary function:		
			FEV1, FVC, FEV1/FVC, MVV	↔ ↔	↔ ↔
			PMS:		
			Handgrip	↔	↔
			1STS	↑ I,C	↑
			BP 50% 1RM	↑ I	↑
			HSQ 50% 1RM	↑ I,C	↑
			Dyspnea: mMRC	↓ I,C	↓
			Fatigue:		
			CFQ-11	↓	↓
			FSS	↓	↓
			Anxiety and depression:		
			GAD-7	↔	↔
			PHQ-9	↔	↓
[45] **Li et al. (2021)**	TELE-PR 3–4 sessions/week, 40–60 min/session, 6 weeks on RehabApp aerobic (walking, treadmill, mild running) 20 min, intensity of 30–40% HRR S1–3 and 40–60% HRR W4–6 minimum 2 days/week breathing control 2 sets of 1 min each, 12 reps, 3–4 days/week thoracic expansion exercises 2 sets each of 1 min, 12 reps, 3–4 days/week low limbs strength training, 2 sets × 3 ex, 1 min/set, 12 reps	Educational counselling: Short session (10 min)	Exercise capacity:		
			6MWT	↔	↑↑
			HRQOL: SF-12	↔	↑ PCS
			Pulmonary function:		
			FEV1, FVC, FEV1/FVC, PEF, MVV	↔ ↔	↔ ↑ MVV
			PMS: Squats	↔	↑
			Dyspnea: mMRC	↓ I	↓
[46] **Vallier et al. (2023)**	TELE-PR 5 sessions/week, 4 weeks 1 session/week of walking, 40 min/session, intensity of 90–100% of the HR reached at a 6MWT conducted before 1 resistance training session/week 3 total sessions of individual strength training via videoconference 1 sophrology session via videoconference	IN-HOSPITAL PR aerobic (cycloergometer) 1 session/week, 1 session = 40 min, intensity of 90–100% HR (determined at a CPET previously carried out) 1 session/week resistance training 3 sessions of group strength training 1 sophrology session	Exercise capacity:		
			6MWT	↑ I,C	↔
			HRQOL: VQ-11	↓ I,C	↔
			Pulmonary function:		
			FEV1, FVC, FEV1/FVC, TLCO	↑ FVC I,C ↑ TLCO I,C	↔ ↔
			Dyspnea: mMRC	↓ I,C	↔
			Fatigue: MFI	↓ C	↓ in favor of C
			PMS:		
			1STS	↑ I,C	↔
			Jump-squats	↑ I,C	↔
[47] **Arora et al. (2025)**	TELE-PR 8 weeks active ROM exercises aerobic breathing exercises incentive spirometry	IN-HOSPITAL PR 2 sessions/week, 45 min—1 h/session, 8 weeks aerobic upper and lower limbs strength training breathing techniques nutritional counselling psychological counselling	Exercise capacity:		
			6MWT	↑ I,C	↔
			Pulmonary function:		
			FEV1, FVC	↑ FVC C	↔
			Dyspnea: mMRC	↔	↔
[48] **del Corral et al. (2025)**	TELE AE + RMT 3 days/week, 8 weeks RMT with Oxygen Dual Valve device, 1 morning session of 20 min, 1 afternoon session of 30 min 1 cycle = 10 reps, 6 cycles in total, 1 min pause between cycles 3 min warm up la 30% MIP/MEP 1 cycle MIP/MEP 50% every 2 min MIP/MEP raised by 10% AE (aerobic) via videoconference 1 session of 50 min, intensity of 60–75% MHR (determined at a CPET previously done), 2 days/week	TELE AE + RMT sham RMT with the same protocol as the I group, but using a sham device (0 cm H2O pressure) AE (aerobic) via videoconference, 1 session = 50 de min, intensity of 60–75% MHR (determined at a CPET previously done), 2 days/week	Exercise capacity:		
			CPET VO_2_max (mL/kg/min)	↑ I,C	↔
			Respiratory muscles function:		
			MIP MEP IME	↑ MIP I, C ↑ MEP I ↑ IME I,C	↑ MIP I vs. C ↑ MEP I vs. C ↑ IME I I vs. C
			HRQOL: EQ-5D-5L Index/VAS	↑ Index I,C + VAS I	↔
			Pulmonary function:		
			FEV1, FVC, FEV1/FVC	↔	↔
			PEF	↑ PEF I	↑ PEF
			TLCO	↑ TLCO I	↔
			PMS:		
			1STS	↑ I,C	↔
			Handgrip	↔	↔
			Anxiety and depression:		
			HADS-A/D	↓ I,C	↔
			HADS total	↓ I,C	↔
[49] **Nagy et al. (2022)**	IMT + manual Diaphragm release training, 6 weeks IMT with Powerbreathe device, 2 sets of 30 dynamic breaths, 1 session = max 4 min, 2 min pause between sessions, intensity of 60% MIP, 3 sessions/week 3 sessions/week DR, 2 sets × 10 deep breaths, 1 min pause between sets, 3 min total time	IMT IMT with Powerbreathe device, 2 sets of 30 dynamic breaths, 1 session = max 4 min, 2 min pause between sets, intensity 60% MIP, 3 sessions/week	Exercise capacity:		
			6MWT	↑ I,C	↑
			Respiratory muscles function: MIP	↑ I	↑
			Dyspnea: mMRC	↓ I,C	↓
			Fatigue: FSS	↓ I,C	↓
[50] **Romanet et al. (2023)**	ETR protocol 2 sessions/weeks, 15–60 min/session, 10 weeks aerobic (cycloergometer) continuous or with intervals, intensity of 60–70%MHR (reached at a 6MWT previously done), initially 15 min and gradually raised 45–60 min upper and lower limbs strength training, 4 sets, 6–12 reps	Classic PR: 2 sessions/week, 1 session = 30 min low-moderate intensity aerobic (cycloergometer) upper and lower limbs strength training RMT balance exercises electrostimulation	HRQOL: SF-12	↔	↑ PCS I vs. C
			Dyspnea: mMRC	↔	↓ I
[51] **Bai et al. (2024)**	PR 3 sessions/week aerobic training, 4 weeks MIIT protocol: 1 session of 8 min at initial intensity of 40% WR max (determined at a CPET previously done), 2 min pause, 4 cycles—>4 sessions at 50% WR max—>4 sessions 55% WR max protocol HIIT: 1 session of 8 min at initial intensity of 40% WR max, 2 min pause, 4 cycles—> 4 cycles of 30 s, 30 s pause, la 80% WR max, 25–30 cycles in total. After 4 the intensity reaches 85% WR, then 90% WR, with the same number and duration of cycles	Educational counselling: WHO Guidelines of rehabilitation and self-management after COVID-19	Exercise capacity: CPET		
			VO_2_max (mL/min)	↑ I	↑
			VO_2_ max(mL/kg/min)	↑↑ I	↑
			VO_2_ max (%)	↑ I	↑
			AT at VO_2_ (mL/min)	↑ I	↑
			AT VO_2_ (mL/kg/min)	↑ I	↑
			AT VO_2_ (%)	↔	↔
			VE (L/min)	↔	↔
			VE/VCO_2_	↔	↔
			HRQOL: SF-12	↔	↔
			Pulmonary function:		
			FEV1, FVC, FEV1/FVC	↔	↔
			Anxiety and depression:		
			GAD-7	↔	↔
			PHQ-9	↓ I	↔
[52] **Besnier et al. (2022)**	CARDIOPULM R 3 session/week, 8 weeks aerobic (cycloerogometer) 30 min, first session 10 min, low intensity (10–11 RPE), W2 20 min, W3–8 30 min strength training 3 sets of 10 reps IMT with Powerbreathe device, 3 sets of 10 breaths, daily breathing techniques (pursed lips, abdominal breathing)	No treatment (patients maintain their daily habits)	Exercise capacity:		
			CPET		
			VO_2_ max (mL/min)	↑	↑
			VO_2_ max (mL/kg/min)	↑	↑
			VO_2_ max (%)	↑	↑
			VO_2_ at VT1 (mL/kg/min)	↑	↑
			VO_2_ a VT2 (mL/kg/min)	↑	↑
			VE/VCO_2_	↑	↑
			Exercise capacity:		
			6MWT	↑	↑
			HRQOL: SF-36	↑ PCS + domains PF, BP, GH, MH ↑ PCS C ↓ MH C	↑ MH I vs. C
			Pulmonary function:		
			FEV1, FVC, FEV1/FVC	↔	↔
			Dyspnea: MRC	↔	↓
			PMS:		
			TUG usual speed	↓	↓
			TUG fast speed	↓	↔
			STS-5	↔	↔

Abbreviations: ↑ = increase, ↓ = decrease, ↔ = no statistically significant change (*p* < 0.05), PR = pulmonary rehabilitation, TELE-PR = tele-pulmonary rehabilitation, CARDIOPULM R = cardiopulmonary rehabilitation, I = intervention group, C = control group, min = minute, w = week, FEV1 = forced expiratory volume in the first second, FVC = forces vital capacity, MVV = maximal voluntary ventilation, PEF = peak expiratory flow, PIF = peak inspiratory flow, TLOO = diffusing capacity of carbon monoxide, MIP = maximal inspiratory pressure, MEP = maximal expiratory pressure, SMIP = sustained maximal inspiratory pressure, IME = inspiratory muscle resistance, EPAP = expiratory positive airway pressure, PEEP = positive end-expiratory pressure, 6MWT = 6-min walk test, CPET = cardiopulmonary exercise test, ISWT = incremental shuttle walk test, VO_2_max = maximal oxygen consumption, AT = anaerobic threshold, VT1 = ventilatory threshold 1, VT2 = ventilatory threshold 2, VE = minute ventilation, HRR = heart rate recovery, RPE = Borg scale rating of perceived exertion, ROM = range of motion, Wrmax = maximal workload, MHR = maximal heart rate, SHR = submaximal heart rate, SF-36 = short-form health survey (36 items), SF-12 = short-form health survey (12 items), PCS = physical component score, MCS = mental component score, EQ-5D-5L = 5-dimension 5-level EuroQol questionnaire, WHOQOL-BREF = World Health Organization Quality of Life questionnaire, HRQOL = health-related quality of life, K-BILD = K-BILD questionnaire for quality of life in pulmonary diseases, IMT = inspiratory muscle training, RMT = respiratory muscle training, mMRC scale = modified Medical Research Council dyspnea scale, BDI = baseline dyspnea index, TDI = transitional dyspnea index, Dyspnoea-12 = 12-item dyspnea questionnaire, GAD-7 = Generalized Anxiety Disorder-7 scale, HADS = Hospital Anxiety and Depression Scale, PHQ-9 = Patient Health Questionnaire-9, BDI* = Beck Depression Inventory, BAI = Beck Anxiety Inventory, PMS = peripheral muscle strength, upper limbs = upper limb, lower limbs = lower limbs, 30/1STS = 30-s sit-to-stand test/1 min, QMVC = quadriceps maximal voluntary contraction, HSQ = half-squat, 1RM = one-repetition maximum, FSS = Fatigue Severity Scale, CFQ-11 = 11-item Chalder Fatigue Scale, MFIS = modified impact of fatigue scale, MFI = multidimensional fatigue inventory, VR = virtual reality.

## Data Availability

The original contributions presented in this study are included in the article/Supplementary Material. Further inquiries can be directed to the corresponding author.

## Supplementary Materials

The following supporting information can be downloaded at: https://www.mdpi.com/article/10.3390/medsci14040443/s1, PRISMA 2020 Checklist.

## Abbreviations

The following abbreviations are used in this manuscript:

PR	pulmonary rehabilitation
COVID-19	Coronavirus disease 19
WHO	World Health Organization
mRNA	messenger ribonucleic acid
EBV	Ebstein–Barr virus
HSV6	Herpes Simplex Virus 6
ACTH	adrenocorticotropic hormone
PTSD	post-traumatic stress disorder
PEM	post-exertional malaise
ENT	ear–nose–throat
ATS	American Thoracic Society
AMPARS	α-amino-e-hydroxy-5-methyl-4-isoxalepropionic acid receptor
ESR	erythrocyte sedimentation rate
CRP	C-reactive protein
PRISMA	Preferred Reporting Items for Systematic Reviews and Meta-Analysis
PROSPERO	International Prospective Register of Systematic Reviews
PEDro	Physiotherapy evidence database
RCTs	randomized controlled trials
TELE-PR	tele-pulmonary rehabilitation
CARDIOPULM R	cardiopulmonary rehabilitation
I	intervention group
C	control group
sess	session
min	minute
w	week
FEV1	forced expiratory volume in the first second
FVC	forced vital capacity
MVV	maximal voluntary ventilation
PEF	peak expiratory flow
PIF	peak inspiratory flow
TLCO	diffusing capacity of carbon monoxide
MIP	maximal inspiratory pressure
MEP	maximal expiratory pressure
SMIP	sustained maximal inspiratory pressure
IME	inspiratory muscle resistance
EPAP	expiratory positive airway pressure
PEEP	positive end-expiratory pressure
6MWT	6-minute walk test
6MWD	6-minute walking distance
CPET	cardiopulmonary exercise test
ISWT	incremental shuttle walk test
VO2max	maximal oxygen consumption
AT	anaerobic threshold
VT1	ventilatory threshold 1
VT2	ventilatory threshold 2
VE	minute ventilation
HRR	heart rate recovery
RPE	Borg scale rating of perceived exertion
ROM	range of motion
Wrmax	maximal workload
MHR	maximal heart rate
SHR	submaximal heart rate
SF-36	short-form health survey (36 items)
SF-12	short-form health survey (12 items)
PCS	physical component score
MCS	mental component score
EQ-5D-5L	5-dimension 5-level EuroQol questionnaire
WHOQOL-BREF	World Health Organization Quality of Life questionnaire
HRQOL	health-related quality of life
K-BILD	K-BILD questionnaire for quality of life in pulmonary diseases
IMT	inspiratory muscle training
RMT	respiratory muscle training
mMRC scale	modified Medical Research Council dyspnea scale
BDI	baseline dyspnea index
TDI	transitional dyspnea index
Dyspnoea-12	12-item dyspnea questionnaire
GAD-7	Generalized Anxiety Disorder-7 scale
HADS	Hospital Anxiety and Depression Scale
PHQ-9	patient Health Questionnaire-9
BDI	Beck Depression Inventory
BAI	Beck Anxiety Inventory
PMS	peripheral muscle strength
upper limbs	upper limbs
lower limbs	lower limbs
30/1STS	30 s sit-to-stand test/1 min
QMVC	quadriceps maximal voluntary contraction
HSQ	half-squat
1RM	one-repetition maximum
FSS	Fatigue Severity Scale
CFQ-11	11-item Chalder Fatigue Scale
MFIS	modified impact of fatigue scale
MFI	multidimensional fatigue inventory
VR	virtual reality
